# Synovitis, Acne, Pustulosis, Hyperostosis, and Osteitis (SAPHO) Syndrome in Childhood; A Rare Clinical Entity

**Published:** 2014-08-15

**Authors:** Ayşe Sayılı, Ozgur Tosun, Nazan Cobanoglu, Nerin Bahceciler Onder, Fusun Baba, Salih Kavukcu

**Affiliations:** 1 Department of Pediatrics; 2 Department of Radiology; 3 Department of Pathology, Near East University Faculty of Medicine, Turkish Republic of Northern Cyprus

**Keywords:** SAPHO Syndrome, Majeed Syndrome, Recurrent Osteomyelitis, Chronic Recurrent Multifocal Osteomyelitis

Dear Editor,

Referring to synovitis, acne, pustulosis, hyperostosis and osteitis; SAPHO syndrome is defined as a chronic, relapsing rheumatologic disease of uncertain etiology characterized by distinct osteoarticular and cutaneous manifestations. There have been recent reports of chronic recurrent multifocal osteomyelitis (CRMO) occurring in adults and SAPHO syndrome occurring in children whereas just the vice-versa is normally expected^[^^1^^,^^2^^]^. Herein, we would like to emphasize a rare form of SAPHO syndrome in terms of age and the localization of the disease.

**Fig. 1:**
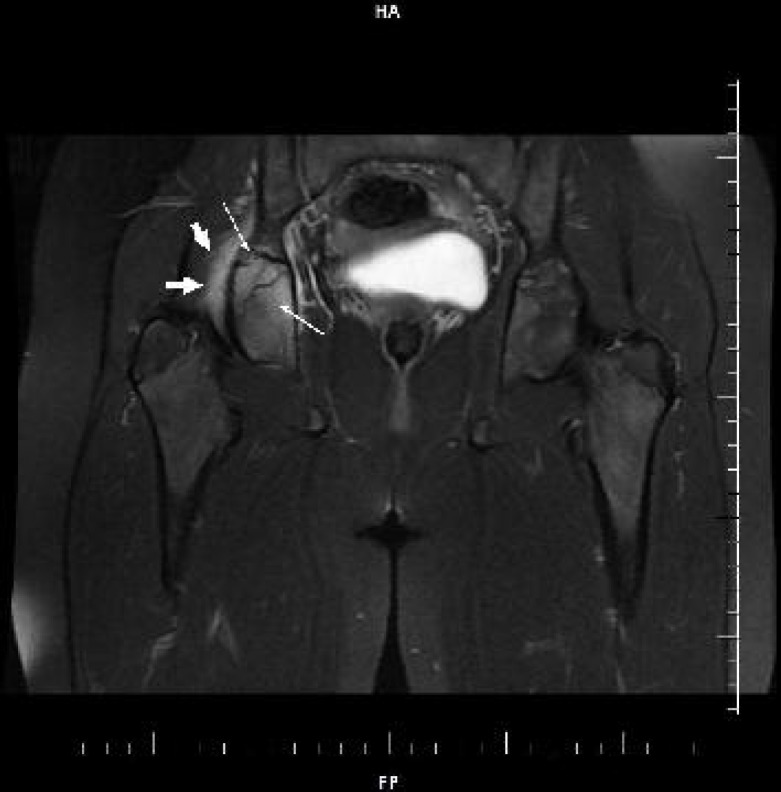
Coronal fat suppressed T2 weighted image shows edema in right acetabulum and surrounding soft tissues

 A 12-year-old female patient was admitted to our hospital with the complaint of right hip and low back pain. She had tenderness on the right iliac crest. She had normal hemogram with an erythrocyte sedimentation rate (ESR) of 51 mm/hour. Serological tests for Salmonella and Brucella were negative. Having a BCG vaccination scar, PPD was negative and no abnormalities were found in chest radiography of the patient. Bone marrow aspiration and bone scintigraphy revealed no signs of malignancy. Anti-streptolysin O, C-reactive protein, anti-nuclear anitobody, anti double stranded DNA and HLA B27 were negative. Serum immunoglobulin levels were within the normal range. Magnetic resonance imaging (MRI) (Fig. 1), showed increased density of right acetabular area and surrounding soft tissue besides bone marrow edema. She received 15 mg/kg/day naproxen sodium administered.

**Fig. 2:**
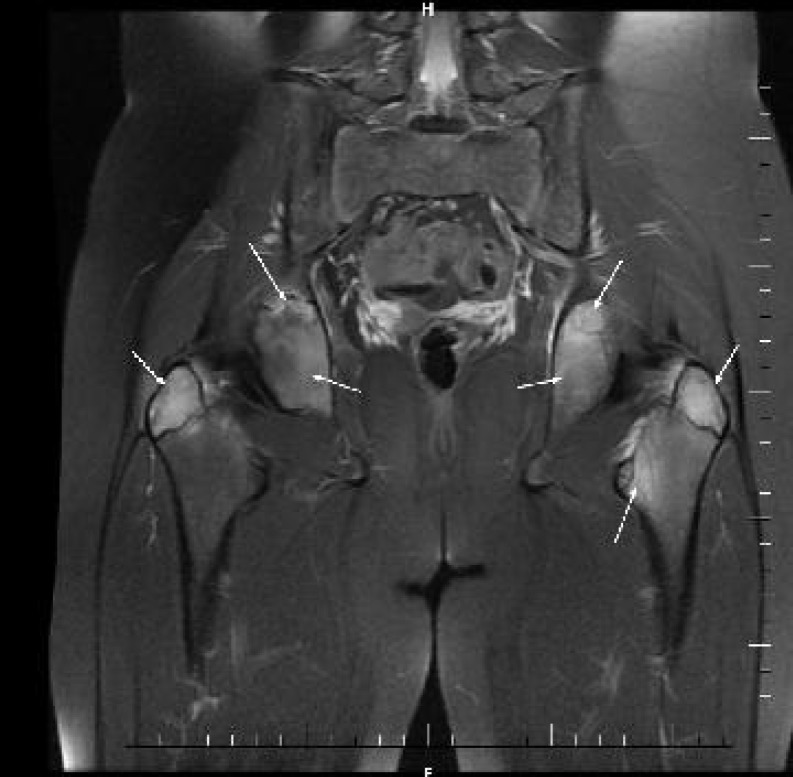
3 months later: coronal fat suppressed T2 weighted image shows edema signal at bilateral acetabulum, major trochanter and left minor trochanter

 In a month’s time, regression of complaints occurred while occasional emergence of fever and pain on the left hip was identified. Repeated pelvic MRI (Fig. 2) revealed involvement of head of femur on the left side, L5 and S1 vertebrates characterized with hyper-density and edema.

**Fig. 3:**
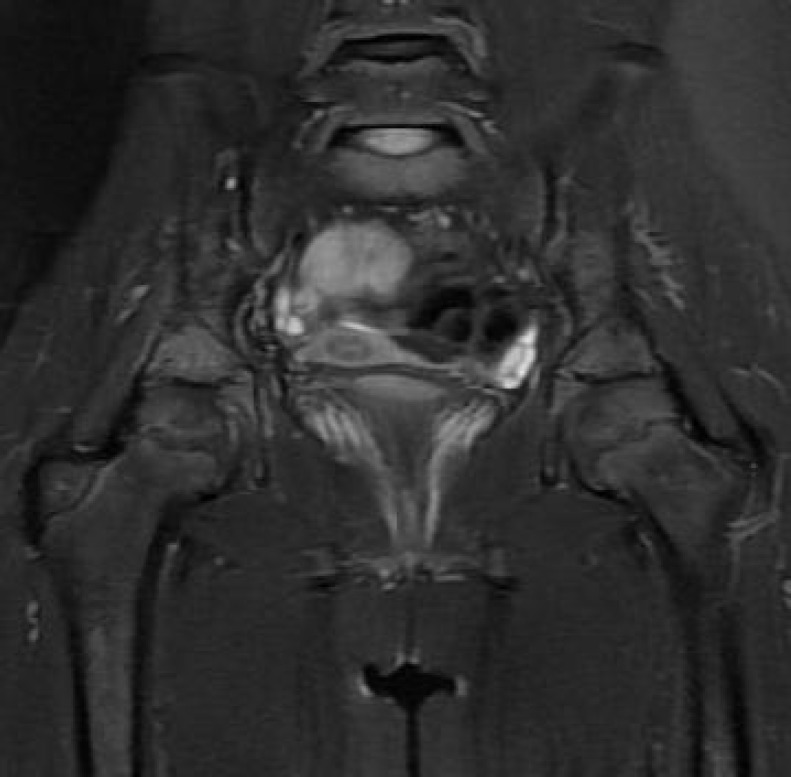
6 months later: signal intensity of edemain. The bilateral acetabulum has decreased, however, there are new edema signals noted in the sacrum

Naproxen sodium treatment was continued for almost next 4 months. She stopped having this treatment on her own accord, and then applied to our hospital with vesiculopustular skin lesions on the palmar, plantar and retroauricular surfaces. Histopathologic findings revealed subcorneal intraepidermal vesicles as well as intracorneal plasma insudation. Lymphocyte exocytosis, infiltrating vesicles and epidermal cells were also visualized. Patient received oral analgesic, non-steroidal anti-inflammatory drug (Ibuprofen) and topical steroid treatment. Remarkable increase in the intensity of edema signals was notable in the evaluation of serial radiological findings (Fig 3). Naproxen sodium administration was continued and after one month there was a clinical regression in the skeletal system findings and skin lesions. Being in the 18^th^ month, she is still under follow up with no problems occurring to date.

 Classification of periodic fever syndromes under the auto-inflammatory disorders has been followed by the definition of auto-inflammatory diseases with particular involvement of bone tissue.

 These are mainly, CRMO, SAPHO syndrome, Majeed syndrome, deficiency of interleukin-1 receptor antagonist (DIRA) and cherubism. SAPHO syndrome is a clinical entity characterized by inflammation in bone, joint and skin. Inflammation

manifests in the form of sterile osteomyelitis and hyperostosis in the bone, acne or pustulosis in the skin and synovitis in the joints.

SAPHO syndrome has been frequently described in adults by rheumatologists, while CRMO has been considered as the form of the disease with similar findings specific to pediatric age groups, particularly 7-12 year olds and mostly amongst girls^[^^4^^]^. 

 In cases with involvement of atypical sites or single skeletal lesions that lack signs of hyperostosis corresponding with radiologic findings and skin manifestations, the diagnosis becomes much more challenging for SAPHO^[^^3^^]^. The vertebral corner involvement in SAPHO syndrome unlike to ankylosing spondylitis, progresses to the adjacent disk space and vertebrae leading prevertebral soft-tissue thickness that supports the diagnosis^[^^4^^]^.

 In CRMO, like SAPHO syndrome, skin manifestations just as described in our patient, are rare findings. Since the skin findings defined pathologically in our patient are characteristic for SAPHO syndrome together with the absence of neutrophil infiltration^[^^5^^,^^6^^]^, we strongly believe that our case has the diagnosis of SAPHO syndrome rather than CRMO.

 The disease course in children just like in adults, is characterized by periods of exacerbations and remissions with an increasing number of lesions over time and considered to be a relatively benign and self-limiting disease without major sequelae^[^^6^^,^^7^^]^.

 Recognition of SAPHO syndrome in our case despite the atypical presentation both in terms of age and skeletal involvement seems notable, since early diagnosis is important to avoid unnecessary invasive procedures and prolonged antibiotic treatment of osteoarticular lesions.

It should be considered in the differential diagnosis of hip and lower back pain. 
